# How Can We Improve the Transformation Success Rate of Research Results in the Pharmaceutical Industry? The Game Theoretic Model of Technology Transfer Subjects

**DOI:** 10.3390/ijerph16091588

**Published:** 2019-05-07

**Authors:** Ziyuan Sun, Man Wang, Weiwei Zhang, Yanli Li, Dan Wang, Feng Dong

**Affiliations:** School of Management, China University of Mining and Technology, Xuzhou 221116, China; wangman1216@163.com (M.W.); zhangweiwei1027@163.com (W.Z.); liliyanyanlili@163.com (Y.L.); wangd9310@163.com (D.W.)

**Keywords:** transformation of pharmaceutical research results, evolutionary game, Stackelberg game, numerical simulation

## Abstract

University–industry technology transfer (UITT) plays an important role in the construction of the national pharmaceutical innovation system. The speculations of a faculty inventor may hinder the successful transfer of pharmaceutical research results. This paper divides the specific process of the transformation of pharmaceutical research results into two parts: (1) an evolutionary game between faculty inventors and universities; and (2) a Stackelberg game between faculty inventors and pharmaceutical companies. Further, we carry out numerical simulations to analyze the impact of transformation success rate, income distribution coefficient, and a faculty inventor’s future working years on the transformation of pharmaceutical research results. The findings indicated that whether a combination of action strategies of faculty inventors and universities can evolve to the optimal equilibrium is determined by many factors, such as the technological transaction price of the pharmaceutical company and the reward or the income obtained by the faculty inventor. The transformation success rate and the income distribution coefficient are the key factors that affect the faculty inventor’s will and the behavior of the pharmaceutical company. The conclusions of this paper contribute to the research on how we can improve the success rate of research results and avoid resource waste, and provide a decision-making reference for the management of pharmaceutical research results in universities.

## 1. Introduction

Technological innovation has become an essential factor influencing economic fluctuations that occur with the continuous improvements in globalization [1]. H Brooks, an American scholar, first defined technology transfer, in 1966, as the process of human activities to disseminate technological results. Nowadays, an innovation linkage and technology transformation mechanism is gradually taking shape in industry as a whole and even in society as a whole. In the field of medicine, the use of basic scientific research to achieve commercially viable technological and pharmaceutical innovations is critical to pharmaceutical progress [2]. According to the World Health Organization, “health technology” refers to “the application of organized knowledge and skills in the form of equipment, drugs, vaccines, procedures, and systems developed to solve health problems and improve the quality of life” [3]. Pharmaceutical research and development is a tedious process; once successful, it will be of great help to future clinical practice. However, the pharmaceutical research results of faculty inventors cannot always be successfully applied to new products. According to Chalmers and Glasziou [4], of the nearly 240 billion U.S. dollars spent on pharmaceutical research annually in the world, up to 85% of the funds are not well-utilized due to inefficiency. Technology transformation is the process of bringing pharmaceutical research results from the laboratory to the market [5]. In this process, the main task of universities is not only teaching and research but also its role in the knowledge production system. This trend is closely related to the increasing penetration of innovation in different sectors of society [6].

In recent years, China has made remarkable achievements in major pharmaceutical research fields. According to the statistics, as of 30 April 2018, China has produced 1800 major pharmaceutical research results. However, less than 144 of them have been transformed, and the transformation success rate is less than 8%. Taking pharmaceutical universities as an example, less than 30% of the total research results are applicable technological achievements, and less than 15% can be popularized. In western developed countries, the conversion rate of R&D technological achievements is as high as 50%, or even 70%. Compared with the more mature mode of the Industry–University–Research combination abroad, there are bottlenecks in the transformation of research patents from Chinese universities and research institutes to the market. The most difficult problem to solve is the valuation of innovative pharmaceuticals in the research phase and the distribution of benefits in the application phase.

**Figure 1:**
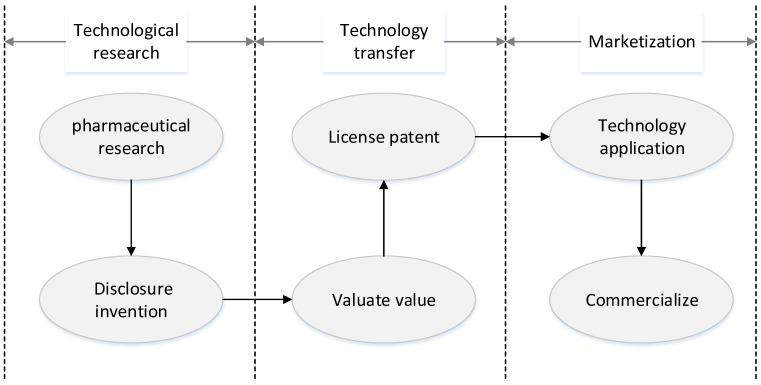
The process of the transfer of pharmaceutical research results.

University–Industry Technological Transformation (UITT) plays an irreplaceable role in the construction of the innovation system and upgrading of the industrial structure in the field of pharmaceuticals. Figure 1 shows the process of the transfer of pharmaceutical research results. Universities are vital links in the Industry–University–Research chain. Achievements provided by faculty have become an essential source of innovation for many companies [7,8]. However, due to the lack of professional technological transformation personnel, an unreasonable faculty assessment and incentive mechanism, faculty inventors’ inability to evaluate research results, and complicated procedures for patent transformation approval, many faculty inventors are reluctant to take the initiative to disclose their research results to schools. However, they tend to cooperate directly with companies or self-created companies to implement external technology transformation [9]. On the one hand, universities lose ownership of such intellectual property rights. Studies have shown that at least 50% of technology transformation occurs when companies cooperate directly with faculty inventors [10]; on the other hand, this speculative behavior of faculty inventors results in poor application of technological results held by universities, and it is difficult to transfer them to pharmaceutical companies. According to licensors’ estimates, 71% of technological results require inventors to work together to be commercialized successfully [11,12,13].

At present, universities need to establish and improve the transformation platform of and center for pharmaceutical research results and actively carry out technological transformation for faculty inventors. In this process, technology authorities in universities should give priority to the establishment of a reasonable regulatory mechanism and a benefit distribution mechanism, and make a reasonable evaluation of the value of pharmaceutical results. This will ensure that universities and pharmaceutical companies obtain benefits and also effectively stimulate the enthusiasm of faculty inventors to participate in the transformation of pharmaceutical results. Taking this into account, game theory is particularly critical in this process. An evolutionarily stable strategy (ESS) of the evolutionary game can help universities and faculty inventors adjust their behavior over time until they maximize their profits. Moreover, the Stackelberg game is a price leadership model that can better describe the relationship between faculty inventors and pharmaceutical companies: faculty inventors as leaders, and pharmaceutical companies as followers.

On the basis of scholars’ research, and considering the moral hazard or adverse selection of faculty inventors in the transformation of existing pharmaceutical results, this paper uses an evolutionary game and a Stackelberg game to study technological transaction prices and the decision-making in transformation channels between the two sides of the game, and further carries out numerical simulations to analyze the transformation success rate of pharmaceutical results, the income distribution coefficient of universities, and the future working life of faculty inventors. The impact of the game equilibrium results is to provide a decision-making reference for the management of pharmaceutical results in universities.

The rest of this paper is arranged as follows. In Section 2, we introduce the current situation of technological transformation in the pharmaceutical field and review the related research. Section 3 points out the methods used in this paper and describes the basic variables and assumptions of the game model of the transformation of pharmaceutical results. Section 4 introduces the evolutionary game model between faculty inventors and universities and carries out numerical simulations based on case studies. Section 5 introduces the Stackelberg game model between faculty inventors and pharmaceutical companies and carries out a simulation and an analysis of the corresponding results. Section 6 discusses related problems in the process of transferring pharmaceutical results. Section 7 draws conclusions and elaborates on future research directions.

## 2. Literature Review

### 2.1. Current Situation of Technological Transformation in the Pharmaceutical Field

By comparing the process of technological transformation in other countries, such as the United States, we can find that developed countries, such as Britain and Germany, realized that university technology is crucial to the development of companies as early as the 1980s and 1990s. For example, the United States has gradually attached importance to the transformation of university technology since the Bayh-Dole Act of 1980 [11]. Policies supporting its drug development industry are attractive to governments [14], focusing on the establishment of research centers for open source pharmaceuticals [15]. As powerful countries in the development of the pharmaceutical industry, developed countries, such as the United States and the United Kingdom, have rich experience in the transformation of pharmaceutical research results. The United States is an important gathering place for the world’s pharmaceutical industry. There are three existing technological transformation modes in its universities. One is the Wisconsin Alumni Research Foundation (WARF), the other is the third-party mode initiated by the Massachusetts Institute of Technology (MIT), and the third is the Stanford University Technology Licensing Office (OTL). At present, the OTL mode is widely used in research universities in the United States. For example, Harvard University, MIT, and the National Institutes of Health in the United States, with the financial support of the Federal Government of the United States, carry out disease mechanism exploration, find drug targets and lead compounds, and then license or transfer technology to pharmaceutical companies. To promote pharmaceutical transformation, European governments established better facilities as early as the 1990s to help pharmaceutical start-ups. For example, clinical trials and their assessments are considered faster and cheaper in Europe than in the United States [16]. Since the 20th century, the British government has continued to intensify the collaborative innovation of industry, education, and research in the field of pharmaceuticals. Since 2010, the U.K. government has invested 2.8 million pounds in the Centre for Innovation in Cell Therapy Technology, attracting the participation of ReNeuron and Vider, the University of London, and the University of Leeds in the integration of scientific research and clinical and commercial needs in regenerative pharmaceuticals.

### 2.2. Decision-Making Behavior of Game Agents in the Process of Technological Transformation

Most scholars pay more attention to the process of technological transformation mainly from the decision-making behavior of faculty inventors, universities, and companies. From the perspective of faculty inventors, inventors generally have moral hazards in the process of technological transformation, and the ownership of an invention is also a key issue for faculty inventors [17]. From the perspective of universities, the commercial practice mode of the technological transformation office (TTO) is mainly transformation-centered [18]. Although more and more universities have invested many resources, such as incubators, in accelerating entrepreneurship and economic development, the influence of the TTO in technological transformation does not necessarily contribute to the formation of new entrepreneurship opportunities [19]. U.K. universities have also proposed University-Industry (UI) interactions for the low productivity and efficiency of the TTO to promote cooperation between universities and companies [20]. From the perspective of companies, technology acquisition can further enhance the competitiveness of companies, while the ability of companies also promotes successful technological transformation [1]. However, the technological transformation modes chosen by companies of different sizes are different [10]. Small companies focusing on technological innovation tend to transfer technology from individuals, while large companies focusing on vertical integration of R&D activities tend to cooperate with universities [10].

### 2.3. Factors Influencing the Technological Transformation Success Rate in the Pharmaceutical Industry

There is much research on the mode of technological transformation in universities, and analyzing the influence of institutional settings, achievement transformation returns, reward mechanisms, and evaluation systems on technological transformation [21,22]. Most scholars believe that creating an appropriate incentive mechanism is the key factor for successful technological transformation. Firstly, universities should decentralize their power appropriately. This not only ensures that researchers and their groups have enough freedom to participate in and operate the technological transformation process, and make rational use of the income, but also stimulates research teams to use research results to develop innovation markets [23,24]. Secondly, because most of the faculty inventors’ technological achievements have not been commercialized, universities should promote cooperation within and among departments, effectively encourage university patent holders, and then increase the number of possibilities for patent licensing [25].

Interaction between universities and pharmaceutical companies is becoming increasingly frequent. Assessment of the value of pharmaceutical patents is equally important for faculty inventors, universities, and companies [26], which has become the primary task of promoting academic research to commercial applications [9]. We also need to pay attention to the TTO, the hub of the transformation of pharmaceutical results in universities, as it not only controls the realization of the benefits of the transformation of pharmaceutical results but also affects the efficiency of it. Siegel et al. [22] found, through 55 interviews, that 55% of managers and entrepreneurs are not satisfied with the marketing and negotiation skills of TTO personnel. The TTO talks with faculty inventors and pharmaceutical companies. These two factors also play a vital role in the process of transformation of pharmaceutical results. There are some key factors: the scale and innovation level of faculty inventors [27,28], the number of postdoctoral and professional researchers, the geographical distance between universities and high-tech companies [29], and the cultural differences between faculty inventors and companies [22]. For example, Wen-Hsiang [30] set multiple variables and sub-variables from three perspectives: technological transferor (university), technological transferee (industry), and technological transformation intermediary structure. The study found that “the ability of the transferor” and “the incentive of the transferor” are equally important, that is, universities should give more incentives to those who have an ability to innovate. Bania et al. [31] found a positive correlation between University R&D and the number of start-ups in the same Standard Metropolitan Statistical Area (SMSA). As for some external factors, pharmaceutical companies may not be able to control them. Therefore, pharmaceutical companies must first solve problems caused by the internal structure and operation, such as improving the ability to adopt the latest pharmaceutical results and narrowing the gap between the R&D and production sectors [32].

Besides this, the successful realization of the transformation of pharmaceutical results depends not only on the correct decision-making and behavior of each subject but also on the support of policy. Strategies and policies to promote the transformation of pharmaceutical results must be adjusted to encourage all subjects to participate in collaborative research actively, and more rigorous laws and regulations are needed to measure the behavior of all parties [33,34].

Although the choice of a faculty inventor’s business behavior is significant in the transformation of pharmaceutical results, the current research only provides a theoretical basis for a governance model of technological transformation from the cooperative path of social subjects. It has not yet touched on the specific supervision mechanism of technological results and the benefit distribution mechanism of universities, nor has it analyzed the impact of these mechanisms on a faculty inventor’s motivation and behavior to participate in the transformation of pharmaceutical results. At present, most universities in China generally adopt the attitude of “not advocating, not encouraging” a faculty inventor’s technological research outcomes and lack effective supervision and control. Therefore, it is necessary to study the process of technological transformation from the perspective of universities’ supervision, and systematically analyze the behavioral characteristics and influencing factors in the process of transformation of pharmaceutical results. The main contributions of this paper are as follows:

- This study is divided into two different game models: faculty inventors and universities, and faculty inventors and pharmaceutical companies, to analyze the game mechanism among the leading players in the process of transformation of pharmaceutical results.
- We studied the ESS for faculty inventors and universities, and analyzed the strategic choices of faculty inventors and pharmaceutical companies, focusing on improving the transformation success rate of the pharmaceutical results.
- We proposed a case study and carried out a data simulation to prove the validity of the two models, and explored the influence of different factors on the transformation success rate of the pharmaceutical results.

## 3. Methodology

### 3.1. Problem Recognition and Description

In recent years, many universities in China have established technology transfer centers and actively cooperated with companies in various fields, such as medical technology research. For example, in June 2018, China Pharmaceutical University entered into a cooperation project with Wanbangde Pharmaceutical Group Co., Ltd. and Zhejiang Shengda Group Co., Ltd. However, the transfer of pharmaceutical research results is not a simple linear process, but a complex nonlinear system. There is a symbiotic relationship among the subjects in the system. They cooperate and compete. Therefore, establishing a sound technology transfer system is of considerable significance to improve the transformation success rate of medical results. However, a successful technology transfer process involves multiple stakeholders, which causes many problems:

(1) The value of medical research results is high, and it is not easy for universities to supervise and track the behavior of faculty inventors. These lead to the existence of “speculative” behavior of faculty inventors, that is, faculty inventors directly contact pharmaceutical companies across universities, or self-run companies to transform their pharmaceutical research results.

(2) Because of the existence of information asymmetry and market competition, universities, faculty inventors, and pharmaceutical companies are eager to maximize their profits through strategy selection.

Under the current university technology management system, faculty inventors should “comply with the rules”, that is, disclose their latest pharmaceutical research results to the university promptly on time, and the university technology transfer department should record and implement result transformation or licensing. However, at this time, the question arises as to how much income will be allocated to faculty inventors and how much reward will be given to faculty inventors, which has become an important reference for faculty inventors to make decisions. Therefore, the interaction between universities and faculty inventors will affect their own strategic choices. Also, for universities and faculty inventors in real economic life, it is tough to achieve complete rationality and complete information. Based on this, the evolutionary game assumes that the participants are bounded and rational, and considers multi-round decision-making interaction between the participants. It simulates the consequences of the players’ strategy selection until the ESS is obtained [35,36].

Faculty inventors and pharmaceutical companies are the suppliers and demanders of research results. No matter what path faculty inventors choose, they should cooperate with pharmaceutical companies in the end. In this process, there are differences in the order of action. Firstly, faculty inventors choose whether to “comply with the rules”. Pharmaceutical companies receive the signals of technology transfer and respond to their preferences and conditions. Then, faculty inventors make strategies that are in line with the overall interests according to these responses. Finally, the transfer of pharmaceutical technology is successful or unsuccessful. The whole process conforms to the hierarchical structure of the Stackelberg game.

### 3.2. Model Establishment

#### 3.2.1. Game Subjects

The transfer mechanism of pharmaceutical research results involves many game players, including faculty inventors, universities, and pharmaceutical companies. We describe the roles of these agents in the technology transfer’s mechanism as follows:

(1) Faculty inventors. Faculty inventors have developed the latest pharmaceutical research results in universities. They can choose to industrialize pharmaceutical research results through technology transfer centers in universities, or they can consult and cooperate with pharmaceutical companies themselves. It depends on the faculty inventors’ assessment of the benefits they can get. Considering the situation of China, once faculty inventors choose to cooperate with universities, they can only continue to abide by this cooperation agreement and will not violate it voluntarily. If faculty inventors do not comply with the agreement, it will be recognized as academic misconduct by universities, seriously affecting their title evaluation and academic development. Faculty inventors will not give up greater benefits in the future for limited benefits in the moment.

(2) Universities. Universities always hope that faculty inventors will disclose the results of pharmaceutical research in a timely manner, and that the technology transfer departments of universities can contact pharmaceutical companies to implement technology transfer or licensing matters. However, universities are not always able to meet expectations, because it is difficult to monitor a faculty inventor’s covert behavior.

(3) Pharmaceutical companies. Pharmaceutical companies aim to achieve the innovation of existing medical resources and conditions by receiving the latest pharmaceutical research results. Successful transformation means that companies can directly apply the research results to the market. If they cannot cooperate with faculty inventors or universities, they can only rely on independent innovation; of course, there is a risk of failure.

This paper mainly discusses the results of pharmaceutical research transferred through cooperation or exchange between pharmaceutical companies and technology transfer institutions set up by universities. In this process, faculty inventors, universities where faculty inventors are located, and companies are usually involved. The following decisions are made in the game process: As the supplier of pharmaceutical research results, faculty inventors have two strategies: “complying with the rules (p)” and “speculation (1-p)”. As the competent department, universities also have two strategies of “non-supervision (q)” and “supervision (1-q)” in the process of technology transfer. Universities also stipulate the income distribution coefficient of faculty inventors and determine the reward given to faculty inventors when they provide pharmaceutical research results to universities. Pharmaceutical companies are the demanders in the technology transfer chain. First, they negotiate a technology transaction price with other parties. When they decide to receive results from outside, they will succeed or fail in technology transfer.

**Figure 2:**
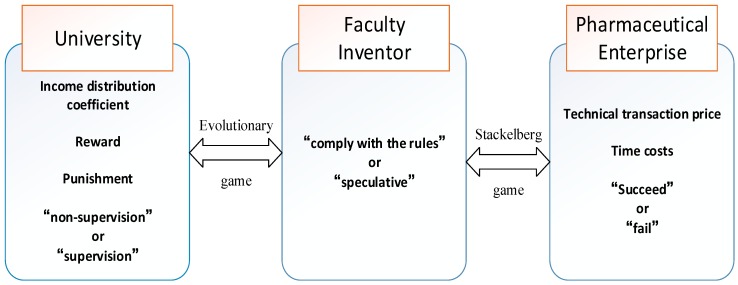
The specific process of the game among universities, faculty inventors, and pharmaceutical Companies.

In the process of transforming pharmaceutical research results, universities and faculty inventors participate in the first round of games, and faculty inventors and pharmaceutical companies participate in the second round of games. Faculty inventors first decide whether to “comply with the rules”, that is, whether to disclose the pharmaceutical research results to universities. If “complying with the rules” is chosen, universities will send a signal of result supply to pharmaceutical companies; if “speculation” is chosen, faculty inventors will directly contact pharmaceutical companies or run their own companies. Pharmaceutical companies analyze the content of the signal and then send the transaction price signal to universities or faculty inventors. Finally, universities or faculty inventors choose the optimal transformation path according to the price signal of pharmaceutical companies. The specific process of the game among universities, faculty inventors, and pharmaceutical companies is shown in Figure 2.

#### 3.2.2. Variables and Hypotheses

Table 1 lists the various symbols used in the article.

Based on the actual situation of the transfer of research results in China’s pharmaceutical industry, we propose the following hypotheses to help construct the model:

**Hypothesis 1** (H1)**.** *Although faculty inventors or universities face multiple pharmaceutical companies in their decision-making processes, the model assumes that they only play games with one company.*

**Hypothesis 2** (H2)**.** *The game subject has symmetric information with no difference, that is, both sides of the game obtain different benefits only because they adopt different behavior strategies, which have nothing to do with their attributes.*

**Hypothesis 3** (H3)**.** *When a faculty inventor “complies with the rules”, the university will accept the results of its pharmaceutical research, and the results must be transferred to serve society.*

**Hypothesis 4** (H4)**.** *The cost of searching for the transfer of pharmaceutical research results by faculty inventors or universities is not considered.*

**Hypothesis 5** (H5)**.** *The probability that a faculty inventor chooses to “comply with the rules” is p. If a faculty inventor “complies with the rules”, the income earned by the faculty inventor is $\gamma p_{s}+T$; otherwise, the full technological transaction price $p_{s}$ is obtained.*

This article assumes that the younger the faculty inventor, the greater the future benefits of the rewards. Consider the use of an annuity to represent college awards, with i for the annual interest rate, $A_{1}$ for each award annuity, and n for a faculty inventor’s future years of work, then the expression for T is $T=A_{1}i^{-1}\left[1-{\left(1+i\right)}^{-n}\right]$.

**Hypothesis 6** (H6)**.** *The probability that a university will not adopt regulatory measures for the transfer of pharmaceutical research results is q. If the university adopts “non-supervision”, a faculty inventor “complies with the rules”, the university’s income function is $\left(1-\gamma \right)p_{s}-T$, a faculty inventor “speculates”, and the college income is 0. If the university supervises the process, a faculty inventor “complies with the rules”, the income function of the university is $\left(1-\gamma \right)p_{s}-C-T$, a faculty inventor “speculates”, and the income of the university is $C_{r}-C$.*

Based on traditional rational thinking, universities are an independent individual in the process of transferring pharmaceutical results. When it is known that a faculty inventor has chosen “speculation”, the economic behavior adopted by universities is an attempt to obtain the greatest economic benefits at their own minimum economic cost. Therefore, the punishment of faculty inventors in universities must be able to make up for the regulatory costs, that is, $C_{r}>C$.

**Hypothesis 7** (H7)**.** *When a faculty inventor “complies with the rules”, the income function of the pharmaceutical company is $p_{12}\pi -p_{s}-C_{t}$, and when a faculty inventor “speculates”, the income function of the pharmaceutical company is $p_{12}\pi -p_{s}$. The failure of transformation is equivalent to independent innovation. At this time, if a faculty inventor “complies with the rules”, the income function of the pharmaceutical company is $p_{11}\pi -S-C_{t}$; and if a faculty inventor is “speculative”, the income function of the pharmaceutical company is $p_{11}\pi -S$.*

## 4. Evolutionary Game between Faculty Inventors and Universities

### 4.1. Evolutionary Game Model

Among the various interest groups, the university or its subordinate technological achievements transformation department is the authority that directly manages the results of faculty inventors’ pharmaceutical research, and the supervision department that transforms the research results. Its management and supervision are prerequisites for the legalization and smooth transformation of pharmaceutical research results in universities. The game between universities and faculty inventors is the first round of the game in the multi-stakeholder game of technology transfer. Therefore, the author first selects universities and faculty inventors as the players in the first round of the game. In this process, by measuring gains and losses, the faculty inventor determines whether he or she will privately resell the pharmaceutical research results of the invention to the enterprise, and the university determines whether to focus on supervising the faculty inventors’ result transformation process.

**Figure 3:**
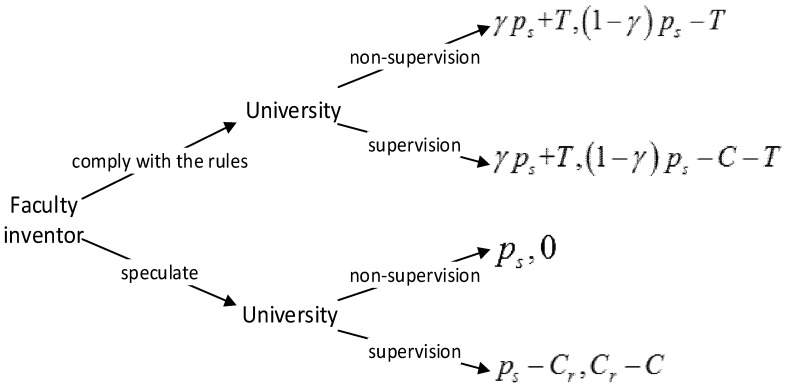
The decision-making tree of the game model between faculty inventors and universities.

Based on the model hypotheses, we establish a game model between universities and faculty inventors, as shown in Figure 3.

The payment matrix is based on different decisions of faculty inventors and universities, as shown in Table 2.

Under the premise that faculty inventors choose “comply with the rules”, when universities adopt the strategy of “non-supervision” and “supervision”, the sum of the faculty inventors’ expected income value is as follows:

(1) $$F_{1}^{1}=\left(\gamma p_{s}+T\right)q+\left(\gamma p_{s}+T\right)(1-q).$$

Under the premise that the faculty inventors choose “speculation”, when universities adopt the strategy of “non-supervision” and “supervision”, the sum of the faculty inventors’ expected income value is as follows:

(2) $$F_{1}^{2}=p_{s}q+\left(p_{s}-C_{r}\right)(1-q).$$

Therefore, the average value of the faculty inventor’s income is:

(3) $$\begin{array}{l} F_{1}=F_{1}^{1}p+F_{1}^{2}\left(1-p\right)\\ =\left[\left(\gamma p_{s}+T\right)q+\left(\gamma p_{s}+T\right)(1-q)\right]p+\left[p_{s}q+\left(p_{s}-C_{r}\right)(1-q)\right]\left(1-p\right) \end{array}.$$

Under the premise that universities choose “non-supervision”, when faculty inventors choose the strategy of “complying with the rules” and “speculation”, the expected return value of universities is:

(4) $$F_{2}^{1}=\left[\left(1-\gamma \right)p_{s}-T\right]p+0*\left(1-p\right).$$

Under the premise that universities choose “supervision”, when faculty inventors choose the strategy of “complying with the rules” and “speculation”, the expected return value of universities is:

(5) $$F_{2}^{2}=\left[\left(1-\gamma \right)p_{s}-C-T\right]p+\left(C_{r}-C\right)\left(1-p\right).$$

Therefore, the average earning value of the university is:

(6) $$\begin{array}{l} F_{2}=F_{2}^{1}q+F_{2}^{2}(1-q)\\ =\left\{\left[\left(1-\gamma \right)p_{s}-T\right]p+0*\left(1-p\right)\right\}q+\left\{\left[\left(1-\gamma \right)p_{s}-C-T\right]p+\left(C_{r}-C\right)\left(1-p\right)\right\}(1-q) \end{array}$$

According to the basic principles of the evolutionary game, from the formulae (1) and (3), the faculty inventor chooses the “complying with the rules” strategy’s duplicate replication dynamic equation:

(7) $$\frac{dp}{dt}=p\left(F_{1}^{1}-F_{1}\right)=p(1-p)\left[T-p_{s}(1-\gamma )+C_{r}\left(1-q\right)\right].$$

According to formulas (4) and (6), the duplicated replication dynamic equation adopted by universities in the “non-supervision” strategy is:

(8) $$\frac{dq}{dt}=q(F_{2}^{1}-F_{2})=q(1-q)\left[C-C_{r}(1-p)\right].$$

Thus, a two-dimensional continuous dynamic system is formed.

By analyzing the local stability of the Jacobian matrix $\varphi =\left(\begin{array}{cc} \frac{\partial \bar{p}}{\partial p} & \frac{\partial \bar{p}}{\partial q}\\ \frac{\partial \bar{q}}{\partial p} & \frac{\partial \bar{q}}{\partial q} \end{array}\right)$ of the system, the stability of the equilibrium point of the two-dimensional continuous dynamic system can be obtained.

Among them, $\frac{\partial \bar{p}}{\partial p}=\left(1-2p\right)\left[T-p_{s}(1-\gamma )+C_{r}\left(1-q\right)\right]$, $\frac{\partial \bar{p}}{\partial q}=C_{r}p(p-1)$, $\frac{\partial \bar{q}}{\partial p}=C_{r}q(1-q)$, $\frac{\partial \bar{q}}{\partial q}=(1-2q)\left[C-C_{r}(1-p)\right]$.

The dynamic system of an evolved population simultaneously satisfies two conditions: $\frac{dp}{dt}=0$, $\frac{dq}{dt}=0$. In combination with the Jacobian matrix of the system, we can obtain five singularities: A(0,0), B(0,1), C(1,0), D(1,1), and E($\frac{C_{r}-C}{C_{r}}$, $\frac{T-p_{s}(1-\gamma )+C_{r}}{C_{r}}$). However, since the specific value of the parameter at point E is not yet determined, it is necessary to discuss the influence of the parameter relationship on the position of point E in different cases.

### 4.2. Equilibrium Analysis

In this system, $C_{r}-C<C_{r}$ must be established, so we compare the size of T, $p_{s}\left(1-\gamma \right)$, $C_{r}$ and carry out a classification discussion. The sufficient condition of stable points in the evolutionary game is the determinant of the Jacobian matrix det(φ)>0 and the trace tr(φ)<0. At this point, the stable point is the local asymptotic stability point of the system, which is the ESS of the evolutionary game.

Case 1: when $C_{r}-C<C_{r}$ and $p_{s}(1-\gamma )-T>C_{r}$. As shown in Table 3, there are four equilibrium points A(0,0), B(0,1), C(1,0), and D(1,1) inside the system. Point A is a stable equilibrium point, C is an unstable equilibrium point, and B and D are the saddle points of the evolutionary game system. In this case, the pharmaceutical company provides higher transaction prices; at the same time, the punishment of the university is insufficient. Speculators appear in the faculty groups to gain more benefits. At this time, universities will intensify their supervision. The result of this dynamic process is that point A appears in the figure.

**Figure 4:**
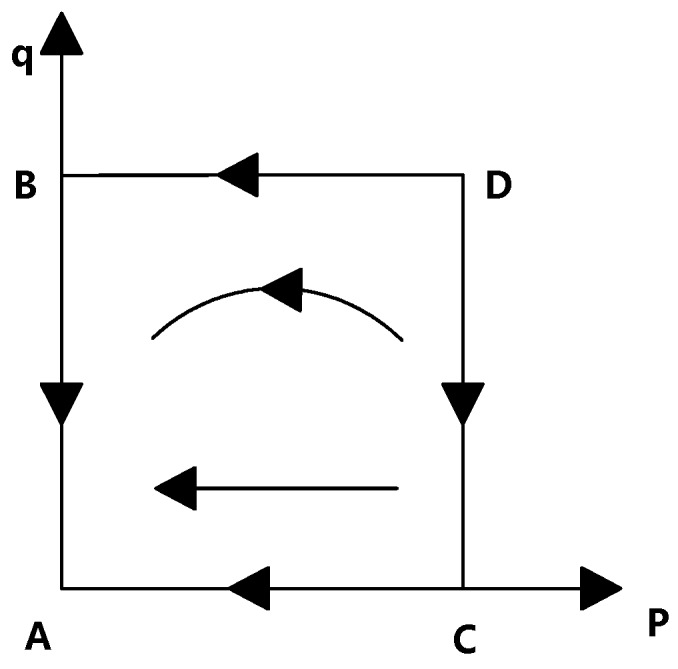
The evolution of phase diagrams in Case 1 ($p_{s}(1-\gamma )-T>C_{r}$).

At the same time, because the universities have allocated too few gains or rewards to faculty inventors, the process of transforming pharmaceutical research results has failed to achieve a fair sense of distribution of interests among universities and faculty inventors, so faculty inventors are more inclined to take the risk of adopting a “speculative” strategy. In the end, as shown in Figure 4, faculty inventors choose to “speculate”, and universities supervise this process.

Case 2: when $C_{r}-C<C_{r}$ and $0<p_{s}(1-\gamma )-T<C_{r}$. In this case, the transaction prices provided by pharmaceutical companies have decreased slightly, and the punishment of universities for faculty inventors who choose to “speculate” has been strengthened. However, the benefits and rewards obtained by faculty inventors for transferring pharmaceutical results through universities have not fully met their expectations. At this time, even if it is possible to face the punishment of universities, risk appetites will still choose “speculation”, while those with risk aversion will be satisfied with the existing income and avoid the punishment and other losses. Therefore, as shown in Table 4, the choice between the two parties is uncertain, and there is no stable point in the system.

Case 3: when $C_{r}-C<C_{r}$ and $p_{s}(1-\gamma )-T<0$. There are four equilibrium points in the system, A(0,0), B(0,1), C(1,0), and D(1,1). Among them, as shown in Table 5, points A and C are the saddle points of the game system, point B is the unstable equilibrium point, and point D is the stable point. Under such circumstances, the transaction price given by the pharmaceutical company is not enough to attract faculty inventors to take risks to “speculate”. It is also necessary that the benefits universities allocate to faculty inventors and the rewards that universities give to faculty inventors meet the faculty’s expectations.

**Figure 5:**
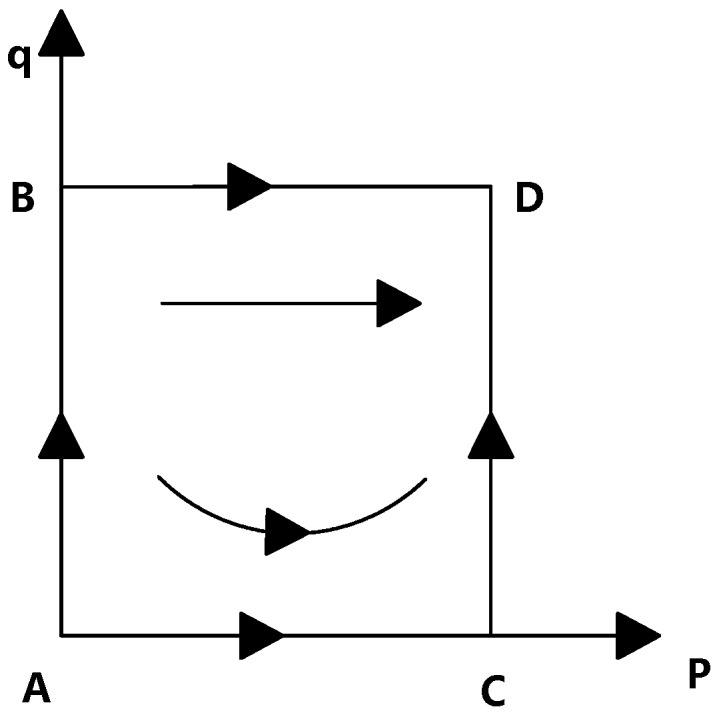
The evolution of phase diagrams in Case 3 ($P_{S}(1-\gamma )-T<0$).

As shown in Figure 5, the evolutionary phase of game dynamics shows that the game process between faculty inventors and universities achieves optimal results: faculty inventors tend to “comply with the rules”, and universities tend to “non-supervise”. At this time, faculty inventors can carry out the transformation of pharmaceutical research results through the technological transformation institutions of universities, and universities choose to trust faculty inventors. Thus, these can save supervision costs, and maximize the benefits of both parties.

To ensure that the game model tends to reach an optimal equilibrium solution, universities need to adjust the corresponding parameters, such as income distribution coefficient and punishment intensity. Several approaches to integration should be used. Firstly, it is appropriate to improve the punishment $C_{r}$ of faculty inventors in universities and reduce the cost of supervision C in universities. The punishment of faculty inventors in universities can effectively limit their “speculation” behavior. Once faculty inventors think that the punishment exerted by universities on “speculative” activities is too great, faculty inventors will voluntarily give up “speculation” and transfer pharmaceutical research results through universities’ intermediaries. In addition, universities must effectively control their supervision costs, and effectively supervise the behavior of faculty inventors to ensure that faculty inventors’ pharmaceutical research results can be successfully transformed. Secondly, universities must increase the faculty inventors’ income distribution coefficient γ and increase the reward T for faculty inventors’ successful transformation. The less the universities give faculty inventors, the more likely faculty inventors are to take greater risks to choose “speculate”. Conversely, if faculty inventors expect to receive more from universities, then their willingness to comply with the rules formulated by universities is stronger.

### 4.3. Case Study and Simulation Analysis

Fudan University is a world-renowned, crucial top-level university in China. It was founded in 1905, and is located in Shanghai, China. The school’s medical department has repeatedly won national science and technology awards. Huya Bioscience International (HUYA) is the leader in globalizing China’s biopharma innovation. HUYA has emerged as the partner-of-choice for maximizing the value of biopharmaceutical innovation from China by developing both early and late-stage drug candidates in concert with our partners primarily in oncology and cardiovascular disease.

In March 2016, Fudan University and HUYA Company reached an agreement in Shanghai. Yang Qing, a professor at the School of Life Sciences of Fudan University, licensed the Indoleamine 2,3 -dioxygenase (IDO) inhibitor with independent intellectual property rights to HUYA Company of the United States. The inhibitor was used for tumor immunotherapy. The transfer of the license brought benefits to both Fudan University and Professor Yang Qing and brought the pharmaceutical technology to HUYA’s clinical trials. We investigated this technology transfer event and estimated the relevant parameters in the two game models for model simulation.

Also, according to the research data obtained after the author visited several key universities, the proportion of transformation income rewarded to faculty inventors (excluding the faculty team and the department) in universities is generally between 0.4 and 0.7 to promote the transformation of technological achievements. A few universities even reached 0.8. Some universities also allocated the transformation income of faculty inventors’ scientific research results according to the university’s horizontal subject management methods. Table 6 lists the distribution of the transformation income for several typical universities [37].

#### 4.3.1. Model Simulation Results

In this section, we obtain the numerical results from the game analysis between universities and faculty inventors and utilize MATLAB to simulate the theoretical analysis. The location of ESS is determined by the parameters of T, Cr, C, Ps, and γ. We assign several parameters with different fixed values, and then change the values of these parameters to observe the changes of ESS according to different hypotheses and situations.

Case 1: based on the hypothesis, the parameters are selected as follows (The selection of variables, such as P_11_, P_12_, π, Cr, Ct, S, A_1_, x, i, and n, is based on the interview content of the subject group, not the empirical data. The related conclusions of the model sensitivity analysis will also be based on this data, and it is not yet effective to prove whether there is any other mutation): T=20, $C_{r}=40$, C=30, $p_{s}=110$, γ=0.4.

**Figure 6:**
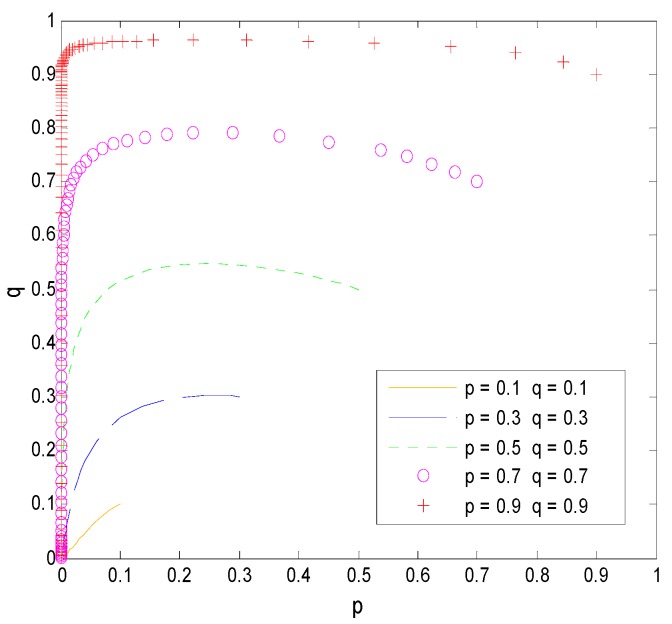
The evolution process of Case 1.

As shown in Figure 6, when $C_{r}-C<C_{r}$ and $p_{s}(1-\gamma )-T>C_{r}$, the system has only one stable point (0, 0) under different initial conditions. The point is, faculty transfer research results directly to pharmaceutical companies, and universities choose to supervise the process, which is consistent with the model conclusions.

**Figure 7:**
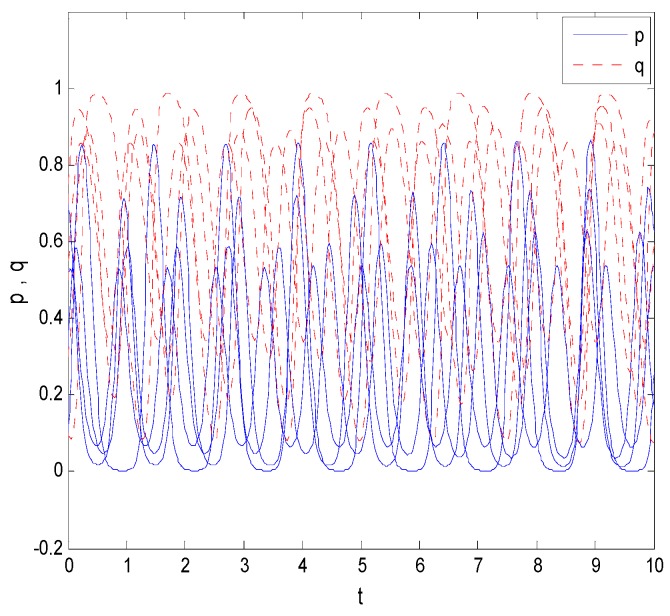
The evolution process of Case 2.

As shown in Figure 7, when $C_{r}-C<C_{r}$ and $0<p_{s}(1-\gamma )-T<C_{r}$, under the conditions of different initial values, the probability that faculty inventors “comply with the rules” and the probability of universities’ supervision of pharmaceutical research results fluctuate up and down, and these probabilities did not tend to a decision group. So, there is no stable point in the system, which is consistent with the model result.

Case 2: based on the hypothesis, the parameters are selected as follows: T=30, $C_{r}=40$, C=30, $p_{s}=90$, γ=0.5.

Case 3: based on the hypothesis, the parameters are selected as follows: T=40, $C_{r}=40$, C=30, $p_{s}=70$, γ=0.7.

**Figure 8:**
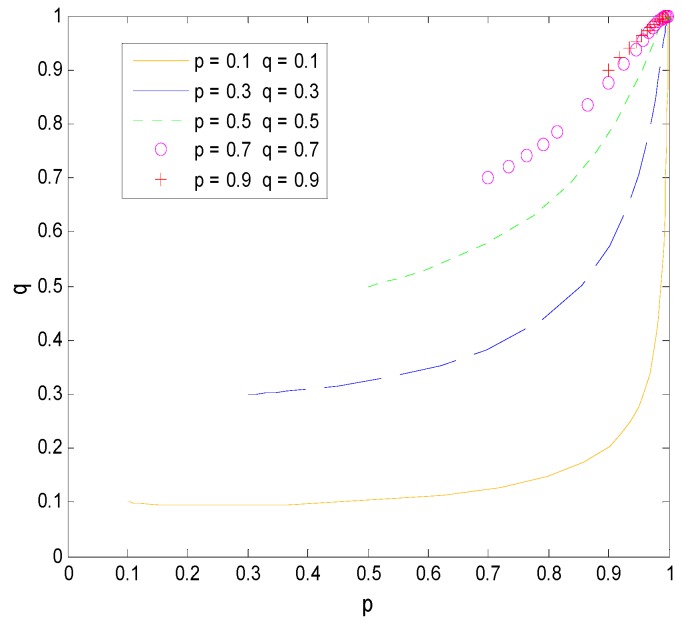
The evolution process of Case 3.

As shown in Figure 8, when $C_{r}-C<C_{r}$ and $p_{s}(1-\gamma )-T<0$, under different initial conditions, the system has only one stable point. Faculty inventors transfer pharmaceutical research results through universities, and universities do not supervise the process, which is consistent with the model conclusions.

#### 4.3.2. Influence of Relative Factors on the Decision-Making of Both Sides of the Game

**Figure 9:**
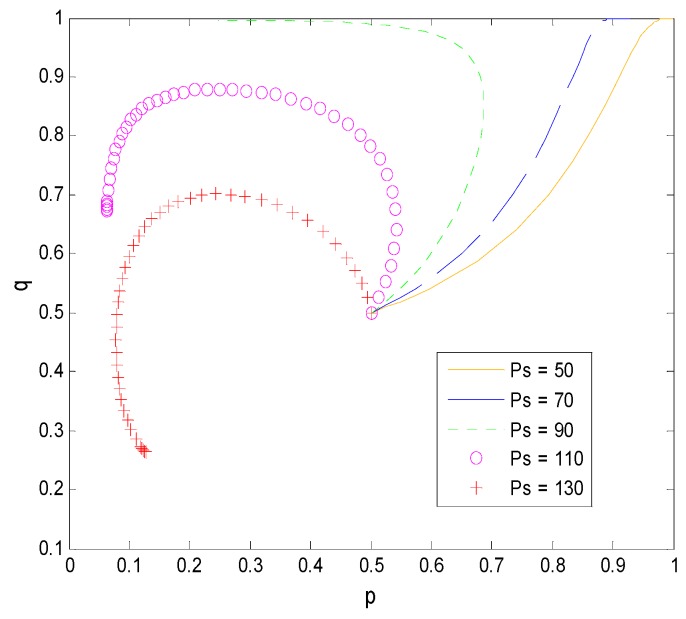
The evolution process of the change of $p_{s}$.

When $p_{s}$ changes, other parameters are selected as follows: T=30, $C_{r}=40$, C=30, γ=0.6. As shown in Figure 9, the system evolves from (1, 1) to the opposite direction as the transaction price provided by the pharmaceutical company is higher when the other parameters are determined. The greater the difference between the benefits of faculty inventors’ direct transfer of pharmaceutical research results to companies and the cost of the risks they bear, the more likely it is that they will choose “speculation”, which is consistent with the actual situation. At the same time, to pursue their own interests and the smooth transfer of pharmaceutical research results, universities will gradually increase their supervision of the technology transfer’s process. Therefore, it is one of the key factors for the success of technology transfer that pharmaceutical companies establish appropriate technology transaction prices.

**Figure 10:**
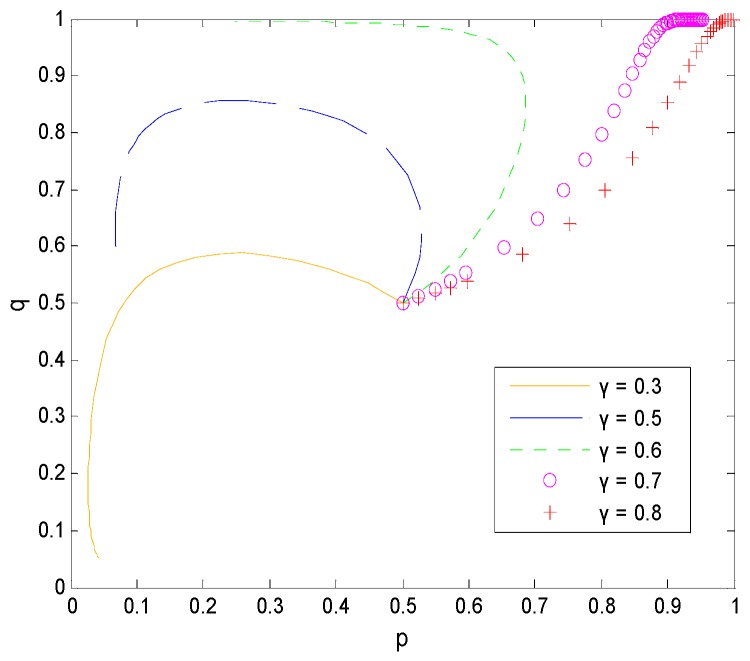
The evolution process of the change of γ.

**Figure 11:**
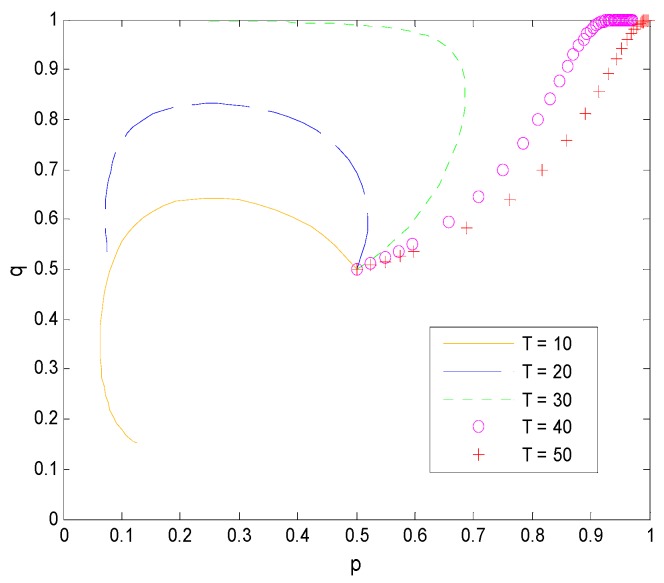
The evolution process of the change of T.

When γ changes, other parameters are selected as follows: $p_{s}=90$, T=30, $C_{r}=40$, C=30. When T changes, other parameters are selected as follows: $p_{s}=90$, γ=0.6, $C_{r}=40$, C=30. From Figure 10 and Figure 11, we can see that the income distribution γ and rewards T given to faculty inventors after the successful transfer of pharmaceutical research results in universities greatly influence the decision-making choices of faculty inventors. As the values of the two gradually increase, the game model gradually evolves from (0, 0) to the optimal equilibrium solution (1, 1). Therefore, compared to simply relying on increased supervision or punishment to prompt faculty inventors to disclose pharmaceutical research results in a timely manner, universities should increase faculty inventors’ income to achieve their expected return value, which can maximize the transformation success rate and promote the effective use of faculty inventors’ research results.

## 5. Stackelberg Game between Faculty Inventors and Enterprises

### 5.1. Stackelberg Game Model

**Figure 12:**
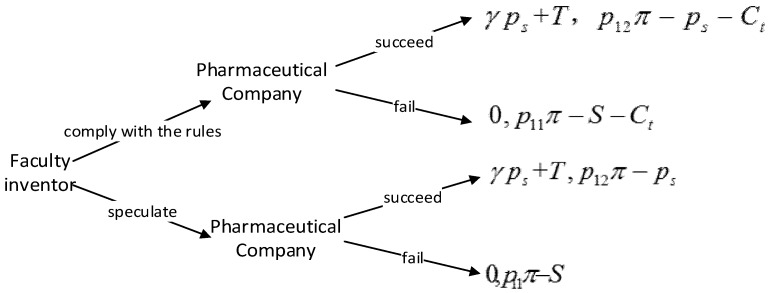
The decision-making tree of the game model between faculty inventors and pharmaceutical companies.

After faculty inventors select “speculation”, they directly send the supply signal of research results to the pharmaceutical companies. Therefore, the inventors are the leaders. Then, pharmaceutical companies measure costs and benefits, and then send signals to the faculty inventors about the transaction price. Thus, pharmaceutical companies are followers. The success of cooperation means that the pharmaceutical research results will be transformed successfully. Conversely, the transformation of pharmaceutical research results will fail. A Stackelberg Game assumes that the leader knows that the follower will react to his decision-making, and the leader also takes the follower’s response into account when making the decision. Moreover, the position of the two in the market may be asymmetric. Therefore, as shown in Figure 12, we establish a Stackelberg game model for faculty inventors and pharmaceutical companies.

When faculty inventors “comply with the rules”, pharmaceutical companies must indirectly obtain pharmaceutical research results from universities; their expected benefits are:

(9) $$\begin{array}{c} {E_{1}}^{1}=\left(p_{12}\pi -p_{s}-C_{t}\right)x+\left(p_{11}\pi -S-C_{t}\right)\left(1-x\right)\\ \;=\left[\left(p_{12}-p_{11}\right)\pi -p_{s}+S\right]x+p_{11}\pi -S-C_{t} \end{array}.$$

When faculty inventors “speculate”, pharmaceutical companies can directly obtain pharmaceutical research results from faculty inventors; their expected benefits are:

(10) $$\begin{array}{cc} {E_{1}}^{2} & =\left(p_{12}\pi -p_{s}\right)x+\left(p_{11}\pi -S\right)\left(1-x\right)\\ & =\left[\left(p_{12}-p_{11}\right)\pi -p_{s}+S\right]x+p_{11}\pi -S \end{array}.$$

Therefore, the expected benefits the pharmaceutical companies receive from the two channels of accepting research results is as follows:

(11) $$\begin{array}{cc} E_{1} & ={E_{1}}^{1}p+{E_{1}}^{2}\left(1-p\right)\\ & =\left[\left(p_{12}-p_{11}\right)\pi +S-p_{s}\right]x+p_{11}\pi -C_{t}p-S \end{array}.$$

The equilibrium condition for a pharmaceutical company to reach a transaction is that the expected return is not less than the independent innovation revenue, so the optimal decision of the pharmaceutical company must meet the following conditions:

(12) $$\begin{array}{l} \underset{p_{s}>0}{\max}E_{1}\left(p_{s}\right)=\left[\left(p_{12}-p_{11}\right)\pi +S-p_{s}\right]x+p_{11}\pi -C_{t}p-S\\ s.t.\begin{array}{cc} \begin{array}{cc} & E_{1}\geq p_{11}\pi -S \end{array} & \end{array} \end{array}.$$

Using the Lagrange method, the transaction price $p_{s}$ should satisfy the following conditions:

(13) $$p_{s}\leq \left(p_{12}-p_{11}\right)\pi +S-\frac{C_{t}p}{x}.$$

In the case of fair competition among a number of pharmaceutical companies, their optimal technology transaction price is:

(14) $$p_{s}^{*}=\left(p_{12}-p_{11}\right)\pi +S-\frac{C_{t}p}{x}.$$

For faculty inventors, the expected benefit of “complying with rules” and “speculation” is:

(15) $$E_{2}=(\gamma p_{s}+T)p+\left(p_{s}-C_{r}\right)(1-p).$$

The result of substituting $p_{s}^{*}$ into formula (15) is:

(16) $$E_{2}=\left(1-p+p\gamma \right)\left[\left(p_{12}-p_{11}\right)\pi +S-\frac{C_{t}p}{x}\right]+Tp-C_{r}(1-p).$$

The optimal probability that faculty inventors “comply with the rules” satisfies the following conditions:

(17) $$\frac{\partial E_{2}}{\partial p}=\left(\gamma -1\right)p_{s}-\frac{C_{t}\left(1-p+p\gamma \right)}{x}+T+C_{r}=0.$$

It can be obtained by the upper form:

(18) $$p^{*}=\frac{x\left(T+C_{r}\right)}{2C_{t}\left(\gamma -1\right)}+\frac{1}{2\left(1-\gamma \right)}+\frac{x\left[S+\left(p_{12}-p_{11}\right)\pi \right]}{2C_{t}}.$$

Putting formula (17) into formula (14), we obtain the following result:

(19) $$\begin{array}{cc} p_{s}^{*} & =\left(p_{12}-p_{11}\right)\pi +S-\frac{C_{t}p}{x}\\ & =\frac{\left(p_{12}-p_{11}\right)\pi +S}{2}+\frac{T+C_{r}}{2\left(1-\gamma \right)}-\frac{C_{t}}{2x\left(1-\gamma \right)} \end{array}.$$

### 5.2. Analysis of Equilibrium Results

In the form of formula (17) and formula (18), the decision behavior of the two parties is influenced by the transformation success rate, the income distribution coefficient, the difference of expected income, and the years of a faculty inventor’s future work and the coefficient of punishment. We will analyze these factors in detail.

**Proposition** **1.** *The relationship between a faculty inventor’s willingness to “comply with rules”, technological transactions prices, and the transformation success rate is as follows:*

*(1) When $0<\gamma \leq 1-\frac{C_{r}+T}{S+(p_{12}-p_{11})\pi }$, a faculty inventor’s willingness to “comply with the rules” is proportional to the success rate of transformation. When $1-\frac{C_{r}+T}{S+(p_{12}-p_{11})\pi }<\gamma \leq 1$, the willingness of faculty inventors to “comply with rules” is inversely proportional to it.*

*(2) Technological transaction prices are directly proportional to the transformation success rate.*

**Proof.**  See Appendix A. □

The relationship between the faculty inventor’s willingness to “comply with the rules” and the transformation success rate is related to such parameters as the income distribution coefficient. Faculty inventors are more willing to “comply with the rules” when the income distribution coefficient is lower than a certain threshold. It is often applied to some pharmaceutical research results that are difficult for individual faculty inventors to transform, such as basic theoretical achievements or applied research results with a low level of innovation. During the transformation of pharmaceutical research results between universities and pharmaceutical companies, faculty inventors tend to “comply with the rules” in this dynamic process, and the probability of successful transformation will increase. Conversely, when the income distribution coefficient is higher than a certain threshold, pharmaceutical research results are easier to transform, and faculty inventors tend to proceed from their own interests and take “speculative” actions. This point was further confirmed by the author’s research on several major universities in China.

Proposition 1 also shows that the price of pharmaceutical research results in technology transactions is positively related to the transformation success rate; the self-interest number is the primary issue for faculty inventors and universities in the process of technology transfer. Therefore, an appropriate increase in technology transaction prices by pharmaceutical companies is conducive to the smooth transformation of research results.

**Proposition** **2.** *The relationship among the coefficient of income distribution and faculty inventor’s willingness to “comply with the rules” and the technological transaction price is as follows:*

*(1) When $0<x\leq C_{t}{\left(C_{r}+T\right)}^{-1}$, the income distribution coefficient is proportional to the faculty inventor’s willingness to “comply with the rules” and inversely proportional to the technological transaction price.*

*(2) When $C_{t}{\left(C_{r}+T\right)}^{-1}<x\leq 1$, the income distribution coefficient is inversely proportional to the faculty inventor’s willingness to “comply with the rules” and proportional to the technological transaction price.*

**Proof.**  See Appendix B. □

Proposition 2 shows that the relationship between the income distribution coefficient and faculty inventor’s willingness to “comply with the rules” and technological transaction prices are determined by the transformation success rate. When the transformation success rate is higher than a certain threshold, research results can be easily transformed by pharmaceutical companies. With an increase in the income distribution coefficient, the price of technological transactions increases, which makes faculty more inclined to obtain all transformation benefits through “speculation”. When the success rate is below this critical point, the opposite is true. It is worth mentioning that the critical threshold is jointly decided by the pharmaceutical companies’ time cost, penalty factor, and the faculty inventor’s rewards of “complying with the rules”.

**Proposition** **3.** *A faculty inventor’s willingness to “comply with rules” and prices of technological transactions are directly proportional to the expected return difference.*

**Proof.**  See Appendix C. □

$p^{*}$ and $p_{s}^{*}$ are proportional to $p_{12}-p_{11}$, which means the greater the expected return difference, the greater the probability that the faculty inventors will “comply with the rules”, and at the same time, the higher the technological transaction price reached by the transferor of research results and pharmaceutical companies.

Proposition 3 also shows that a large difference in expected return means that there is a certain degree of technical gap between faculty inventors and pharmaceutical companies, and the ability of enterprises to undertake technology is lower. Under such circumstances, the technological transformation offices or the faculty inventors themselves will take more energy to help the pharmaceutical companies undertake research results. Therefore, the price of technological transactions will inevitably increase.

**Proposition** **4.** *The willingness of faculty inventors to “comply with the rules” is inversely proportional to their future working years.*

**Proof.**  See Appendix D. □

Proposition 4 shows that a faculty inventor’s willingness to “comply with the rules” changes in the opposite direction of their future working years. The younger faculty inventors are, the more years of future work they have, and the more they tend to be “speculative”. Faculty inventors who teach at a higher age will pay more for a “speculative” behavior. For example, their reputation established over time may be destroyed.

**Proposition** **5.** *The penalties faced by faculty inventors are inversely proportional to their willingness to “comply with the rules” and are proportional to the prices of technological transactions.*

**Proof.**  See Appendix E. □

Proposition 5 indicates that, on the one hand, with the enhancement of supervision and tracking of faculty inventors’ inventions in universities, the cost of universities in the supply of pharmaceutical research results will increase, and the expected benefit will increase accordingly. At this time, universities have requirements for technological transaction prices. If the effect of research results on the income of the pharmaceutical company is limited, the company may reject the university’s price requirements, which in turn reduces the transformation success rate of pharmaceutical research results.

On the other hand, with the increase in transaction prices, faculty inventors will risk being punished and choose to have direct contact with pharmaceutical companies or to run businesses themselves. Under such circumstances, higher penalty rates in universities cannot effectively restrain faculty inventors’ “speculation”.

### 5.3. Numerical Simulation Results

To analyze the effect of such parameters as the transformation success rate, income distribution coefficient, and expected return difference on a faculty inventor’s willingness to “comply with the rules” and technological transaction prices, other parameters, in this case, are assumed to be $P_{11}=0.4$, $P_{12}=0.8$, π=250, $C_{r}=40$, $C_{t}=40$, S=10, T=30, $A_{1}=1$, x=0.7, i=0.01, and n=30. In this section, we also use MATLAB to simulate and support the game-theoretical analysis. The effects of these factors on the decision-making behavior of faculty inventors and pharmaceutical companies are discussed below.

#### 5.3.1. Influence of x on the Decision-Making Behavior of Both Sides of the Game

**Figure 13:**
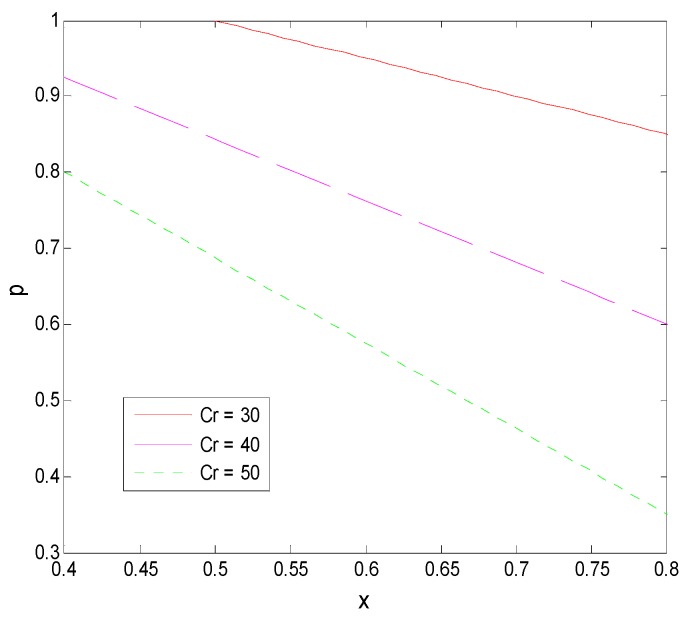
The effect of the transformation success rate on a faculty inventor’s willingness to “comply with the rules”.

From Proposition 1 and Figure 13, we can see that, first, the transformation success rate of pharmaceutical research results in universities is negatively related to a faculty inventor’s willingness to “comply with the rules”, that is, most pharmaceutical research results easily transformed are directly transformed by faculty inventors through “speculative behavior”. This directly leads to the unsuccessful transformation of pharmaceutical research results held by universities and to a low transformation rate of research results in universities. Second, when universities increase the penalty factor to ease the “speculative” behavior of faculty inventors, a faculty inventor’s willingness to “comply with the rules” decreases, demonstrating that it does not fundamentally curb the “speculation” of faculty inventors. Especially in this example, as the transformation success rate and the penalty factor increase together, in the end, only less than 30% of the faculty inventors are willing to “comply with the rules”. This shows that universities should not use the means of increasing the penalty coefficient to control the path of the transformation of pharmaceutical research results, as the penalty coefficient has a minimal effect on the behavior of faculty inventors who have research results with a high transformation success rate.

**Figure 14:**
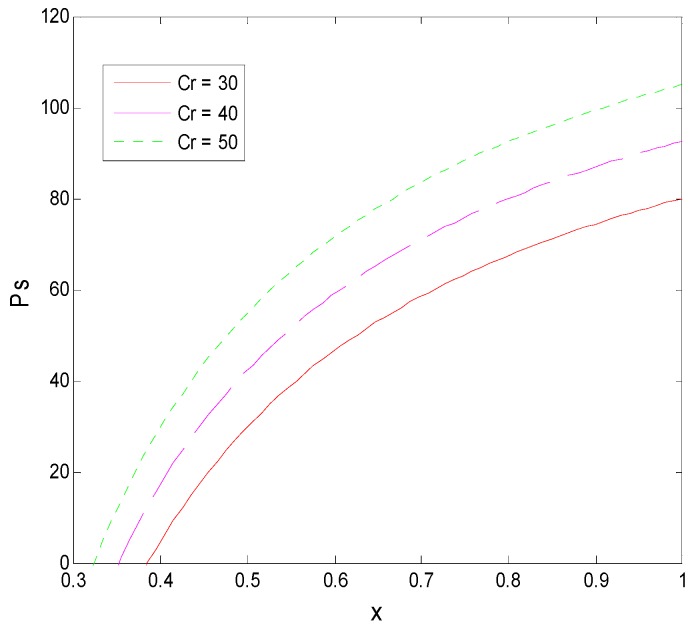
The effect of the transformation success rate on the technological transaction price.

As shown in Propositions 1 and 5 and Figure 14, the price of technological transactions is positively correlated with the transformation success rate and the penalty coefficient of the university. For a pharmaceutical research result that is relatively easy to industrialize, improving the supervision of faculty inventors’ inventions by the technological management department not only cannot effectively restrain faculty inventors’ “speculation”, but will have a certain negative impact on the acceptance of research results by pharmaceutical enterprises because they indirectly increase the price of technological transactions. However, on the other hand, a faculty inventor’s inventions are state-owned assets, and the “inaction” of universities will lead to a large loss of state-owned assets. Therefore, from the perspective of social services, universities should maintain a balance between protecting state-owned assets and promoting the transformation of pharmaceutical research results. Additionally, universities can maintain appropriate supervision, especially to focus on the supervision of major pharmaceutical research results, and actively promote the full-scale development of the transformation of pharmaceutical research results.

#### 5.3.2. Influence of γ on the Decision-Making Behavior of Both Sides of the Game

**Figure 15:**
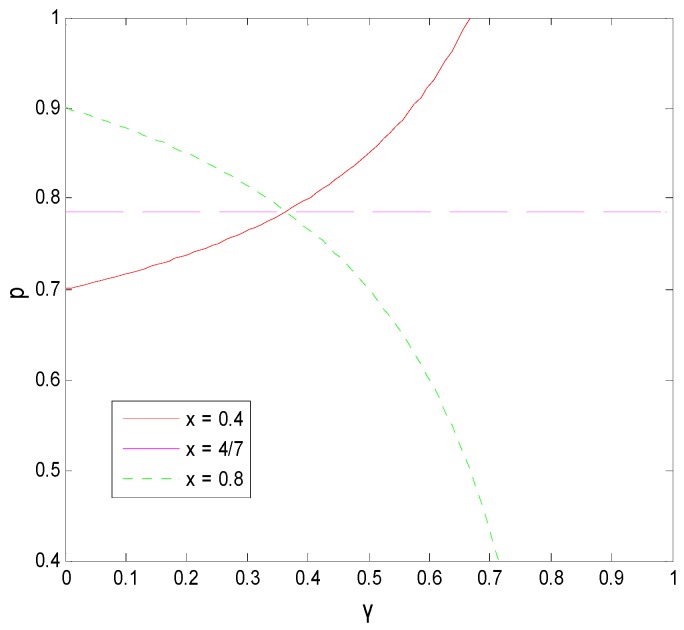
The effect of the income distribution coefficient on faculty inventors’ will to “comply with rules”.

**Figure 16:**
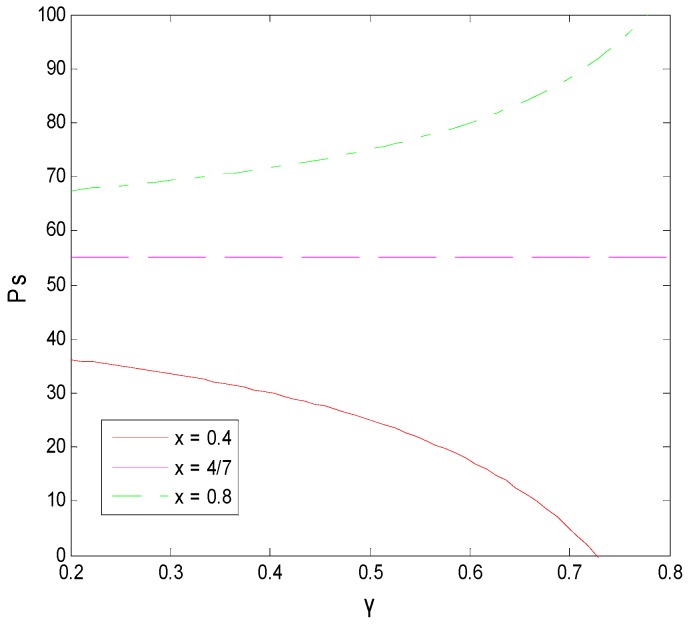
The effect of the income distribution coefficient on the technological transaction prices.

The effect of the income distribution coefficient on the faculty inventor’s willingness to “comply with the rules” and the technological transactions price depends on the success rate of transformation. From Figure 15 and Figure 16, we can see that when x is at the critical point of 4/7 in this example, the income distribution coefficient has little to no effect on the technological transaction price and the faculty inventor’s willingness to “comply with the rules”. However, when x > 4/7, that is, the pharmaceutical research results are easy to transform, the technological transactions price will increase, and the increase rate will be faster than the growth rate of transformation income provided by universities, and thus faculty inventors are more inclined to obtain all transformation profits through “speculation”. x < 4/7 means that the success rate of pharmaceutical research results is low, and the transaction price is generally low. In this case, faculty inventors tend to “comply with the rules” as the income distribution coefficient increases.

It is worth mentioning that the critical point depends on the penalty coefficient, the time cost, and the rewards that faculty inventors receive to “comply with the rules”. Therefore, as the decision-maker of the income distribution coefficient, universities must comprehensively consider the influence of these three factors on the faculty inventor’s willingness to “comply with the rules”. This can help to make decisions that are most conducive to the transformation of pharmaceutical research results. For applied pharmaceutical research results with a high transformation success rate, universities should reduce their income distribution coefficients. However, for basic research results with a low transformation success rate, universities should maintain high-income distribution coefficients to encourage faculty inventors’ scientific research and “comply with the rules”, while avoiding excessive loss of state-owned assets.

#### 5.3.3. Influence of Other Parameters on the Game Model

**Figure 17:**
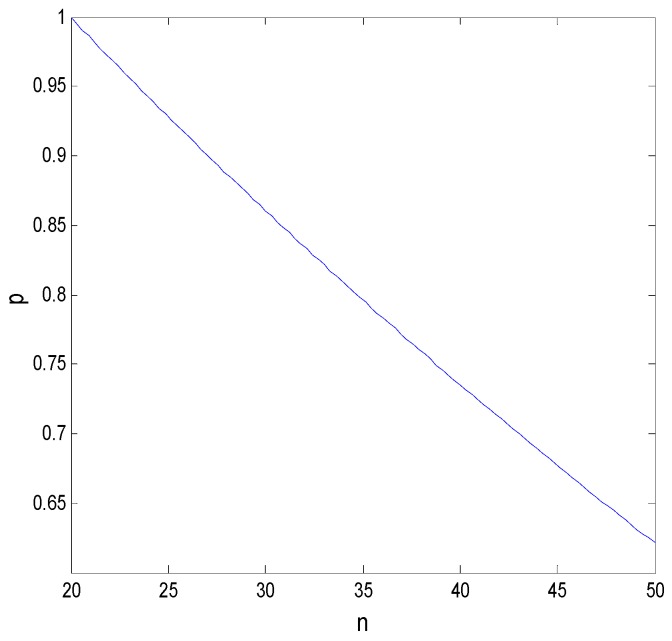
The effect of faculty inventors’ working years on their willingness to “comply with the rules”.

Assume that the income distribution coefficient is 0.6 and the transformation success rate is an equilibrium value of 4/7. In combination with Proposition 4 and Figure 17, faculty inventors will generally choose to “comply with the rules” when their future working life is 20 years. The shorter the faculty inventor’s future working time is, the more inclined they are to complete the transformation of pharmaceutical research results through the technology transfer offices of universities. This can, to a certain extent, reduce the time cost of faculty inventors’ successful transformation of research results, and at the same time, the penalties faced in the case of supervision in universities are avoided. However, when faculty inventors are relatively young, they have more energy and enthusiasm to choose different approaches to technology transfer. At this time, the probability of “speculation” has increased to obtain greater benefits.

**Figure 18:**
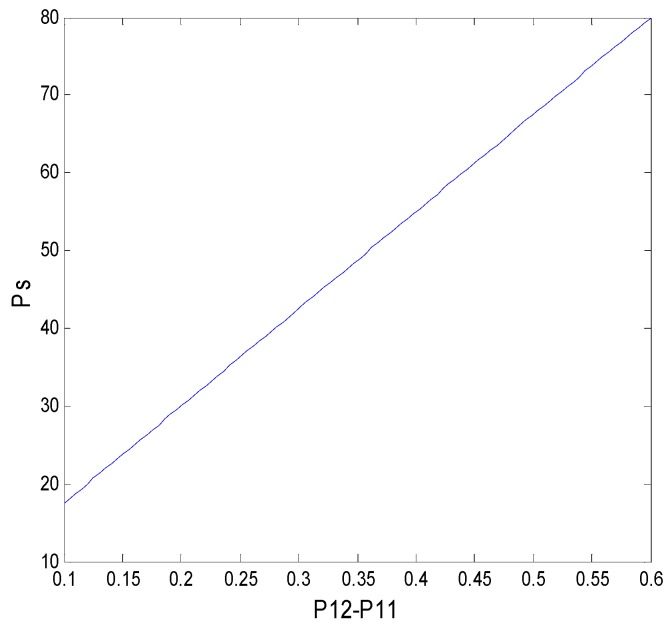
The effect of the expected return difference on the technological transaction prices.

On the other hand, we can see from Proposition 3 and Figure 18 that there is a linearly increasing relationship between the technological transaction prices and the expected return difference. The expected return difference represents the contribution of the faculty inventors’ research results to the pharmaceutical company, that is, the technical gap between the faculty inventor and the pharmaceutical company. The larger the value, the greater the technological transaction price that the pharmaceutical company needs to pay.

## 6. Results and Discussion

The successful transformation of pharmaceutical achievements represents smooth cooperation among game players. Undoubtedly, due to the asymmetric information, the parties cannot accurately identify the intentions of others [38]. In order to ensure the smooth progress of cooperation, the commitment agreement was born [39,40]. In view of the transfer process of pharmaceutical results, the transformation agreement defines and standardizes the behavior of faculty inventors, universities, and pharmaceutical enterprises, and enables participants to understand their expectations and possible consequences [41,42]. So who should draft and pay to set up this enforceable transformation agreement? Through our research on several key universities in China, we find that universities are usually the sponsors of the contract for the transformation of pharmaceutical results. Because universities have abundant scientific research power and a large number of patents, compared with the huge profits they get when the results are successfully transferred, the cost of establishing an agreement is not too high.

A good agreement can promote the collaborative behavior of participants [43,44]. The signing of a transformation agreement is not only decided by one party, but also a product after the participants consult and weigh the interests of all parties. However, people are always willing to make promises when future earnings reach their expectations. Faculty inventors, universities, and pharmaceutical enterprises are eager for the results of the agreement to be more beneficial to their own interests. For example, faculty inventors hope to transfer pharmaceutical results at the highest possible price, universities hope to obtain more transfer income on the basis of ownership of pharmaceutical results, and pharmaceutical enterprises hope to obtain the right to use the results at the lowest price.

Therefore, whether the agreement can be reached and whether the pharmaceutical results can be transformed smoothly mainly depends on the subjective considerations of game players. The above theoretical model illustrates the equilibrium of faculty inventors and universities participating in the evolutionary game and the optimal combination of strategies of faculty inventors and pharmaceutical companies participating in the Stackelberg game. The model also analyzes the impact of such factors as the transformation success rate, the income distribution coefficient, and the expected return difference in the decision-making behavior of the game subjects. Through further numerical simulations, the aforementioned results are demonstrated in the light of actual conditions. This section provides more suggestions for research through the discussion of practical issues, which in turn promote the orderly progress of technology transfer in the pharmaceutical industry.

(1) How can we make faculty inventors choose to “comply with the rules” rather than “speculate”?

The primary consideration of faculty inventors’ participation in technology transfer is whether they choose to actively disclose their pharmaceutical research results to universities. Compared with the previous theoretical studies [45,46], this article provides a more comprehensive understanding of faculty inventors’ behavior choice. According to the results of the first round of the game and the actual situation, we believe that the combination of faculty inventors’ “complying with the rules” and “non-supervision” of universities can maximize their benefits. However, the actual situation is not perfect. Faculty inventors will choose to “speculate” because of the mismatch between their benefits and costs, and their behavior is largely influenced by the universities and pharmaceutical companies. To this end, we analyze the behavioral characteristics of faculty inventors and select the best strategies for faculty inventors to “comply with the rules”.

First, what is the main reason that faculty inventors choose to engage in “speculation”? Jensen and Thursby [47] suggested that the research results are more likely to be applied when the inventor’s return is linked to the licensee’s earnings. However, the authors also pointed out that they did not consider the case of the inventor founding a business in the process of research. To address the moral hazard of inventors, the most common practice in University licensing is to let inventors start their own development company and commercial inventions [17]. In terms of our conclusions, we find that when pharmaceutical research results are more easily transferred, faculty inventors may be more inclined to “speculate”. That is, faculty inventors are more likely to “comply with the rules” for research results that are more difficult to transfer. In other words, the higher the price of technological transactions provided by pharmaceutical companies, the greater the probability of “speculation” by faculty inventors. This shows that faculty inventors are still benefiting groups.

Second, will faculty inventors give up “speculation” because they are afraid of supervision and punishment strategies in universities? Based on the analysis of the equilibrium results and numerical simulations, supervision and punishment do not become obstacles to “speculation” as expected. On the contrary, for the same pharmaceutical research results, the punishment is increased, and the probability of faculty inventors’ “complying with the rules” will be greatly reduced. This shows that the punitive policy has not played a real role.

According to the principles of incentive compatibility [48], we can use incentives to encourage faculty inventors to “comply with rules”, such as increasing faculty inventors’ benefits in technology transfer and giving them material rewards or spiritual support. As a result, the next two issues are worth discussing.

(2) How should universities formulate relevant rules for the transformation of pharmaceutical research results?

Many research conclusions indicate that the attitudes and policies of universities for faculty inventors’ research results influence the behavior of faculty inventors to a certain extent, and in turn determine whether faculty inventors’ research results can be transformed and used effectively [49,50,51]. In research on evolutionary game theory, scholars have explored the effects of punishment and reward on positive behavioral compliance [52,53]. For example, Sigmund et al. [54] stated that, in the process of promoting cooperation between the two sides of the game, rewards and punishment are effective. Balliet et al. [55] obtained the same result. High penalties are often considered as an important way to promote cooperation [56,57]. Hauert et al. [58] argued that cooperation supported by punishment is more likely to be achieved than the compulsory union. In the evolutionary game between universities and faculty inventors, we also deem that the improvement of rewards and punishments promotes the evolution of the decision-making behavior of both sides to the optimal equilibrium solution.

However, when we incorporated pharmaceutical companies into the model, things seem to change. The results show that an income distribution coefficient that is too low and excessive punishment hinder the transformation of pharmaceutical research results. Therefore, in the process of promoting faculty inventors to “comply with the rules”, we should focus on the impact of the income distribution coefficient. When the transformation success rate is lower than a certain critical point, the more the universities improve the income distribution coefficient, the higher the faculty inventors’ probability of “complying with the rules”. However, when the transformation success rate is higher than this critical point, a higher income distribution coefficient of universities has the opposite effect. Therefore, universities need to appropriately adjust the income distribution coefficient according to the specific conditions of pharmaceutical research results to ensure that the interests of faculty inventors reach their expectations, and to maximize the transformation rate of faculty inventors’ pharmaceutical research results through intermediary institutions in universities.

(3) What is the impact of the technological transaction price set by the pharmaceutical company on this process?

In actual corporate strategic positioning and market competition, continuous innovation is indispensable in responding to the rapidly changing economic environment [59]. Highly innovative pharmaceutical companies can often identify and quickly seize new market opportunities and achieve greater profits. These companies tend to have a positive attitude toward innovation and risk-taking, and they will determine whether to conduct research new medicine and how to obtain pharmaceutical research results through the measurement of corporate capabilities, patient demand, market opportunities, competitors, and other comprehensive factors. Many studies show that partnerships between universities and companies affect the process of technology transfer [60,61]. Therefore, as a technology transferee, pharmaceutical companies determine the price of technology transactions, which greatly affects the subjective choice of faculty inventors.

When pharmaceutical research results are more easily transformed or when there is a large gap between faculty inventors and pharmaceutical companies’ technique level, pharmaceutical companies will actively or passively increase transaction prices to promote the successful implementation of technology transfer. However, when the technical gap between faculty inventors and pharmaceutical companies is small, the transaction price agreed by the company is also small. When universities supervise and track faculty inventors’ inventions to a high degree, as shown in Proposition 5, the penalties faced by faculty inventors are directly proportional to the prices of technological transactions. That is, the stronger the supervision of universities, the greater the pharmaceutical company’s cost to gain access to research results, leading some pharmaceutical companies to not accept research results in consideration of cost–benefit issues, which makes it difficult to widely use the pharmaceutical patent held by faculty inventors, causing a waste of resources. As a result, pharmaceutical companies should improve their ability to undertake and to a certain extent reduce the price of technological transactions.

We know that preclinical research, clinical trial research, approval and production, and marketing are the stages for the industrialization of new drug research and development. In this process, clinical trial research, as the most important part, is divided into Phase I, Phase II, and Phase III clinical trials. If the test does not achieve the desired effect, the pharmaceutical research results will not enter the market. As the main person in charge of medical clinical trials, pharmaceutical companies need to invest a lot of human and material resources in this step to ensure the smooth application of results. Therefore, pharmaceutical companies should optimize the proportion of cost investment in the research and industrialization of a new drug, and use more resources for experimental research instead of simply increasing the price of technological transactions.

## 7. Conclusions

In this paper, an evolutionary game model and a Stackelberg game model are used to discuss decision-making in the transformation of pharmaceutical research results between faculty inventors and universities and faculty inventors and pharmaceutical companies. We found that, with the reduction of the technological transaction price, the increase of faculty inventors’ income and rewards, and other related indicators, the strategic combination of faculty inventors and universities can eventually evolve to the optimal equilibrium point that maximizes the interests of both sides. In addition, through a game analysis and numerical simulation between faculty inventors and pharmaceutical companies, we have found that the main factors affecting technology transfer in universities are the transformation success rate, the income distribution coefficient, and the penalty factor. The transformation success rate is inversely proportional to faculty inventors’ willingness to “comply with the rules” and is proportional to the technological transaction price. The effect of the income distribution coefficient on faculty inventors’ “complying with the rules” and technological transaction prices depends on the transformation success rate. Faculty inventors’ future years of work are inversely proportional to their willingness to “comply with the rules”. The penalty factor and the difference between expected returns are directly proportional to the technological transaction price. At the same time, increasing the punishment of universities will further reduce the faculty inventors’ willingness to “comply with the rules” and increase the prices of technological transactions.

However, it is generally difficult to judge the transformation success rate of pharmaceutical research results in real life before technological transactions are achieved, which affects the strategic choice of participants in technology transfer. Moreover, the game model in this paper only studies the impact of the important parameters directly related to the game subject’s strategy, and other important factors are not taken into account. Thus, the strategic behavior space of this model is relatively limited. In addition, innovation is produced in the interaction between universities, companies, and governments [62]. Although universities play a more important role [63], the government’s policy support cannot be ignored either.

Therefore, future research can further consider the impact of other factors on the decision-making of faculty inventors, universities, and pharmaceutical companies in technology transfer, such as incomplete information, risk preference, the achievement transfer contract at the industrial level, the patent ownership system, service invention identification, and the performance evaluation system for universities at the government level. We can further bring the government structure into the model and construct a game decision-making model for the transformation of universities’ pharmaceutical research results with multi-subject participation.

## Figures and Tables

**Table 1:** Variables in the game model.

Symbol	Description
p	Probability of a faculty inventor’s “complying with the rules”, p>0
q	Probability of supervising faculty inventors’ technology transfer behavior in universities, q>0
$p_{s}$	Technological transaction prices provided by pharmaceutical companies to faculty inventors or universities, $p_{s}>0$
γ	The proportion of total income paid by pharmaceutical companies allocated to faculty inventors in universities when faculty inventors “comply with the rules” (abbreviated as the “income distribution coefficient”), γ>0
T	When faculty inventors “comply with the rules”, universities give rewards to faculty inventors after a successful technology transfer (such as a title evaluation or academic awards), T>0
C	The cost of supervising the transfer process of pharmaceutical research results in universities, C>0
$C_{r}$	The punishment for faculty inventors’ “speculation” when discovered by universities (including acceptance of fines, damage to reputation, and other forms of punishment; in this model, these are converted into the number of fines), $C_{r}>C>0$
$P_{11}$	The probability of successful innovation of pharmaceutical companies, $P_{11}>0$
$P_{12}$	The probability that a pharmaceutical company will successfully accept a technology transfer, $P_{12}>P_{11}>0$
π	Total revenue after pharmaceutical companies gain research results, π>0
$C_{t}$	When a pharmaceutical company obtains research results from a university, the time cost $C_{t}$ will be met regardless of success or failure. Because a faculty inventor’s invention is a state-owned asset, and the approval procedures are complicated, $C_{t}>0$
S	The loss of opportunity for pharmaceutical companies who fail to accept a technology transfer but with a successful acceptance by competitors, S>0
x	The probability of successful transfer of pharmaceutical research results, referred to as the “transformation success rate”, x>0

**Table 2:** Variables in the game model.

Faculty Inventors	Universities	
	Non−Supervision (q)	Supervision (1−q)
Comply with the rules (p)	$\gamma p_{s}+T$ $(1-\gamma )p_{s}-T$	$\gamma p_{s}+T$ $(1-\gamma )p_{s}-C-T$
Speculate (1−p)	$p_{s}$ 0	$p_{s}-C_{r}$ $C_{r}-C$

**Table 3:** Stability analysis of Case 1.

Balance Point	det(φ)	tr(φ)	Local Stability
**A (0,0)**	+	−	ESS
**B (0,1)**	−	±	Saddle Point
**C (1,0)**	+	+	Unstable point
**D (1,1)**	−	±	Saddle Point

**Table 4:** Stability analysis of Case 2.

Balance Point	det(φ)	tr(φ)	Local Stability
**A (0,0)**	−	±	Saddle Point
**B (0,1)**	−	±	Saddle Point
**C (1,0)**	−	±	Saddle Point
**D (1,1)**	−	±	Saddle Point
$E(\frac{C_{r}-C}{C_{r}}$ $\left(\frac{T-p_{s}(1-\gamma )+C_{r}}{C_{r}}\right)$	−	0	Saddle Point

**Table 5:** Stability analysis of Case 3.

Balance Point	det(φ)	tr(φ)	Local Stability
**A (0,0)**	−	±	Saddle Point
**B (0,1)**	+	+	Unstable point
**C (1,0)**	−	±	Saddle Point
**D (1,1)**	+	−	ESS

**Table 6:** The income distribution coefficient of several typical universities.

University	Inventor (Team)	University	Department
**Fudan University**	50%	40%	10%
**Zhejiang University**	70%	20%	10%
**Southeast University**	40%	40%	20%
**Tongji University**	50–80%	20–50%	-
**South China University of Technology**	40%	40%	20%

## Appendix A

**Proof of Proposition 1.** The following results can be obtained for the derivative of the transformation success rate x for formula (17):

(A1) $$\frac{\partial p^{*}}{\partial x}=\frac{S+\left(p_{12}-p_{11}\right)\pi }{2C_{t}}-\frac{T+C_{r}}{2C_{t}\left(1-\gamma \right)}$$

Thus, when $0<\gamma \leq 1-\frac{C_{r}+T}{S+(p_{12}-p_{11})\pi }$, $\frac{\partial p^{*}}{\partial x}>0$; When $1-\frac{C_{r}+T}{S+(p_{12}-p_{11})\pi }<\gamma \leq 1$, $\frac{\partial p^{*}}{\partial x}<0$. The following results can be obtained for the derivative of the transformation success rate x for formula (18):

(A2) $$\frac{\partial p_{s}^{*}}{\partial x}=\frac{C_{t}}{2x^{2}(1-\gamma )}>0$$

Thus, $p_{s}$ and x are directly proportional to each other.

## Appendix B

**Proof of Proposition 2.** The following results can be obtained for the derivative of the income distribution coefficient γ for formula (17) and (18):

(A3) $$\frac{\partial p^{*}}{\partial \gamma }=\frac{1}{2{\left(1-\gamma \right)}^{2}}\left[1-\frac{x\left(T+C_{r}\right)}{C_{t}}\right]$$

(A4) $$\frac{\partial p_{s}^{*}}{\partial \gamma }=\frac{1}{2{\left(1-\gamma \right)}^{2}}\left(C_{r}+T-\frac{C_{t}}{x}\right)$$

Thus, when $x\leq C_{t}{\left(C_{r}+T\right)}^{-1}$, $\partial p^{*}/\partial \gamma \geq 0$, $\partial p_{s}^{*}/\partial \gamma \leq 0$; when $x>C_{t}{\left(C_{r}+T\right)}^{-1}$, $\partial p^{*}/\partial \gamma <0$, $\partial p_{s}^{*}/\partial \gamma >0$.

## Appendix C

**Proof of Proposition 3.** The following results can be obtained for the derivative of the expected return difference $p_{12}-p_{11}$ for formula (17) and (18):

(A5) $$\frac{\partial p^{*}}{\partial \left(p_{12}-p_{11}\right)}=\frac{\pi x}{2C_{t}}>0$$

(A6) $$\frac{\partial p_{s}^{*}}{\partial \left(p_{12}-p_{11}\right)}=\frac{\pi }{2}>0$$

Thus, $p^{*}$ and $p_{s}^{*}$ are proportional to $p_{12}-p_{11}$.

## Appendix D

**Proof of Proposition 4.** The following results can be obtained for the derivative of faculty inventors’ future working years n for formula (17):

(A7) $$\frac{\partial p^{*}}{\partial n}=\frac{\partial p^{*}}{\partial T}\frac{\partial T}{\partial n}=\frac{xA_{1}ni^{-1}{\left(1+i\right)}^{-n-1}}{2C_{t}\left(\gamma -1\right)}<0$$

Thus, the probability of the faculty inventors’ “complying with the rules”p is inversely proportional to the faculty inventors’ working years n in the future.

## Appendix E

**Proof of Proposition 5.** The following results can be obtained for the derivative of the penalty factor Cr for formula (17) and (18):

(A8) $$\frac{\partial p^{*}}{\partial C_{r}}=\frac{x}{2C_{t}\left(\gamma -1\right)}$$

(A9) $$\frac{\partial {p_{s}}^{*}}{\partial C_{r}}=\frac{1}{2\left(1-\gamma \right)}$$

Thus, $\partial p^{*}/\partial C_{r}<0$, $\partial {p_{s}}^{*}/\partial C_{r}>0$.

## References

[1] Siriram R., Snaddon D.R. (2004). Linking technology management, transaction processes and governance structures. Technovation.

[2] Van Norman G.A., Eisenkot R. (2017). Technology Transaction: From the Research Bench to Commercialization: Part 1: Intellectual Property Rights—Basics of Patents and Copyrights. JACC BasicTransl. Sci..

[3] MacNeil M., Koch M., Kuspinar A., Juzwishin D., Lehoux P., Stolee P. (2019). Enabling health technology innovation in Canada: Barriers and facilitators in policy and regulatory processes. Health Policy.

[4] Chalmers I., Glasziou P. (2009). Avoidable waste in the production and reporting of research evidence. Obstet. Gynecol..

[5] Mendes P. (2002). Transfusion medicine technology transaction: Traps to avoid. Transfus. Med. Rev..

[6] Rasmussen E., Moen Ø., Gulbrandsen M. (2006). Initiatives to promote commercialization of university knowledge. Technovation.

[7] Dosi G., Llerena P., Labini M.S. (2006). The relationships between science, technologies and their industrial exploitation: An illustration through the myths and realities of the so-called ‘European Paradox’. Res. Policy.

[8] Geuna A., Nesta L. (2006). University patenting and its effects on academic research: The emerging European evidence. Res. Policy.

[9] Sohn S.Y., Lee W.K., Ju Y. (2013). Valuing academic patents and intellectual properties: Different perspectives of willingness to pay and sell. Technovation.

[10] BodasFreitas I.M., Geuna A., Rossi F. (2013). Finding the right partners: Institutional and personal modes of governance of university–industry interactions. Res. Policy.

[11] Colyvas J., Crow M., Gelijns A., Mazzoleni R., Nelson R.R., Rosenberg N., Sampat B.N. (2002). How do university inventions get into practice?. Manag. Sci..

[12] Thursby J.G., Thursby M.C. (2004). Are faculty critical? Their role in university–industry licensing. Contemp. Econ. Policy.

[13] Agrawal A. (2006). Engaging the inventor: Exploring licensing strategies for university inventions and the role of latent knowledge. Strateg. Manag. J..

[14] Lockhart M.M., Babar Z.-U.-D., Carswell C., Garg S. (2013). New Zealand’s Drug Development Industry. Int. J. Environ. Res. Public Health.

[15] Federsel H.-J. (2010). Process R&D under the magnifying glass: Organization, business model, challenges, and scientific context. Bioorgan. Med. Chem..

[16] Gurău C., Dana L., Lasch F. (2012). Academic entrepreneurship in UK biotechnology firms: Alternative models and the associated performance. J. Enterp. Communities People Places Glob. Econ..

[17] Dechenaux E., Thursby J., Thursby M. (2011). Inventor moral hazard in university licensing: The role of contracts. Res. Policy.

[18] Weckowska D.M. (2015). Learning in university technology transfer offices: Transactions-focused and relations-focused approaches to commercialization of academic research. Technovation.

[19] Markman G.D., Phan P.H., Balkin D.B., Gianiodis P.T. (2005). Entrepreneurship and university-based technology transfer. J. Bus. Ventur..

[20] Chau V.S., Gilman M., Serbanica C. (2017). Aligning university–industry interactions: The role of boundary spanning in intellectual capital transfer. Technol. Forecast. Soc..

[21] Friedman J., Silberman J. (2003). University technology transfer: Do incentives, management, and location matter?. J. Technol. Transfer.

[22] Siegel D.S., Waldman D., Link A. (2003). Assessing the impact of organizational practices on the relative productivity of university technology transfer offices: An exploratory study. Res. Policy.

[23] Debackere K., Veugelers R. (2005). The role of academic technology transfer organizations in improving industry science links. Res. Policy.

[24] Macho-Stadler I., Pérez-Castrillo D. (2010). Incentives in university technology transfers. Int. J. Ind. Organ..

[25] Drivas K., Economidou C., Karamanis D., Zank A. (2016). Academic patents and technology transfer. J. Eng. Technol. Manag..

[26] Holmes J.S. (2009). Societal and economic valuation of technology-transfer deals. Acta Astronaut..

[27] Powers J.B. (2003). Commercializing Academic Research: Resource Effects on Performance of University Technology Transfer. J. High. Educ..

[28] O’Shea R.P., Allen T.J., Chevalier A., Roche F. (2005). Entrepreneurial orientation, technology transfer, and spin-off performance of US universities. Res. Policy.

[29] Anselin L., Varga A., Acs Z. (1997). Local Geographic Spillovers Between University Research and High Technology Innovations. J. Urban Econ..

[30] Lai W.-H. (2011). Willingness-to-engage in technology transfer in industry–university collaborations. J. Bus. Res..

[31] Bania N., Eberts R.W., Fogarty M.S. (1993). Universities and the Startup of New Companies: Can We Generalize from Route 128 and Silicon Valley?. Upjohn Work. Pap. J. Art..

[32] Hering S., Loretz B., Friedli T., Lehr C.-M., Stieneker F. (2018). Can lifecycle management safeguard innovation in the pharmaceutical industry?. Drug Discov. Today.

[33] Perkmann M., King Z., Pavelin S. (2011). Engaging excellence? Effects of faculty quality on university engagement with industry. Soc. Sci. Electr. Publ..

[34] Bruneel J., D’Este P., Salter A. (2010). Investigating the factors that diminish the barriers to university–industry collaboration. Res. Policy.

[35] Imhof L.A., Nowak M.A. (2006). Evolutionary game dynamics in a Wright-Fisher process. J. Math. Biol..

[36] Elsadany A.A. (2017). Dynamics of a Cournot duopoly game with bounded rationality based on relative profit maximization. Appl. Math. Comput..

[37] Fong P.S.W., Chang X., Chen Q. (2018). Faculty patent assignment in the Chinese mainland: Evidence from the top 35 patent application universities. J. Technol. Transf..

[38] Han T.A., Santos F.C., Lenaerts T., Pereira L.M. (2015). Synergy between intention recognition and commitments in cooperation dilemmas. Sci. Rep..

[39] Axelrod R., Hamilton W.D. (1981). The evolution of cooperation. Science.

[40] Han T.A., Pereira L.M., Santos F.C. Intention Recognition, Commitment and the Evolution of Cooperation. Proceedings of the 2012 IEEE Congress on Evolutionary Computation.

[41] Wooldridge M., Jennings N.R. (1999). The Cooperative Problem-Solving Process. J. Logic. Comput..

[42] Han T.A., Pereira L.M., Santos F.C., Lenaerts T. (2013). Good Agreements Make Good Friends. Sci. Rep..

[43] Cherry T.L., Mcevoy D.M. (2013). Enforcing Compliance with Environmental Agreements in the Absence of Strong Institutions: An Experimental Analysis. Environ. Resour. Econ..

[44] Han T.A., Pereira L.M., Lenaerts T. (2016). Evolution of Commitment and Level of Participation in Public Goods Games. Auton. Agent. Multi-Ag..

[45] Owen-Smith J., Powell W.W. (2001). To Patent or Not: Faculty Decisions and Institutional Success at Technology Transfer. J. Technol. Transf..

[46] Boh W.F., De-Haan U., Strom R. (2016). University technology transfer through entrepreneurship: Faculty and students in spinoffs. J. Technol. Transf..

[47] Jensen R., Thursby M. (2001). Proofs and Prototypes for Sale: The Licensing of University Inventions. Am. Econ. Rev..

[48] De Castro L., Yannelis N.C. (2018). Uncertainty, efficiency and incentive compatibility: Ambiguity solves the conflict between efficiency and incentive compatibility. J. Econ. Theory.

[49] Chang X., Chen Q., Fong P.S.W. (2015). Scientific disclosure and commercialization mode selection for, university technology transfer. Sci. Public Policy.

[50] Shane S., Dolmans S.A.M., Jankowski J., Reymen I.M.M.J., Romme A.G.L. (2015). Academic entrepreneurship: Which inventors do technology licensing officers prefer for spinoffs?. J. Technol. Transf..

[51] Battaglia D., Landoni P., Rizzitelli F. (2017). Organizational structures for external growth of University Technology Transfer Offices: An explorative analysis. Technol. Forecast. Soc..

[52] Szolnoki A., Perc M. (2008). Coevolution of teaching activity promotes cooperation. New J. Phys..

[53] Hilbe C., Traulsen A. (2012). Emergence of responsible sanctions without second order free riders, antisocial punishment or spite. Sci. Rep..

[54] Sigmund K., Hauert C., Nowak M.A. (2001). Reward and punishment. Proc. Natl. Acad. Sci. USA.

[55] Balliet D., Mulder L.B., Van Lange P.A.M. (2011). Reward, Punishment, and Cooperation: A Meta-Analysis. Psychol. Bull..

[56] Han T.A., Lenaerts T. (2016). A synergy of costly punishment and commitment in cooperation dilemmas. Adapt. Behav..

[57] Boyd R., Gintis H., Bowles S., Richerson P.J. (2003). The evolution of altruistic punishment. Proc. Natl. Acad. Sci. USA.

[58] Hauert C., Traulsen A., Brandt H., Nowak M.A., Sigmund K. (2007). Via Freedom to Coercion: The Emergence of Costly Punishment. Science.

[59] Huang Q., Chen X., Zhou M., Zhang X., Duan L. (2019). How Does CEO’s Environmental Awareness Affect Technological Innovation?. Int. J. Environ. Res. Public Health.

[60] Berbegal-Mirabent J., Sánchez García J.L., Ribeiro-Soriano D.E. (2015). University–industry partnerships for the provision of R&D services. J. Bus. Res..

[61] Thorburn L. (2000). Knowledge Management, Research Spinoffs and Commercialization of R&D in Australia. Asia-Pac. J. Manag. Res. Innov..

[62] Marques J.P.C., Caraça J.M.G., Diz H. (2006). How can university–industry–government interactions change the innovation scenario in Portugal?—The case of the University of Coimbra. Technovation.

[63] Etzkowitz H., Leydesdorff L. (1997). Introduction to special issue on science policy dimensions of the Triple Helix of university-industry-government relations. Sci. Public Policy.

